# LincRNA-p21: Implications in Human Diseases

**DOI:** 10.3390/ijms160818732

**Published:** 2015-08-11

**Authors:** Sai-Sai Tang, Bi-Ying Zheng, Xing-Dong Xiong

**Affiliations:** 1 Institute of Aging Research, Guangdong Medical University, Dongguan 523808, China; E-Mail: saisaitang1988@126.com; 2 Institute of Biochemistry & Molecular Biology, Guangdong Medical University, Zhanjiang 524023, China; 3 Key Laboratory for Medical Molecular Diagnostics of Guangdong Province, Guangdong Medical University, Dongguan 523808, China; 4 Institute of Laboratory Medicine, Guangdong Medical University, Dongguan 523808, China; E-Mail: zby88kr@163.com; 5 Department of Pharmacology and the Penn State Hershey Cancer Institute, Milton S. Hershey Medical Center, the Pennsylvania State University College of Medicine, Hershey, PA 17033, USA

**Keywords:** long non-coding RNA, lincRNA-p21, gene regulation, diseases

## Abstract

Long noncoding RNAs (lncRNAs), which lack significant protein-coding capacity, regulate various biological processes through diverse and as yet poorly understood molecular mechanisms. However, a number of studies in the past few years have documented important functions for lncRNAs in human diseases. Among these lncRNAs, lincRNA-p21 has been proposed to be a novel regulator of cell proliferation, apoptosis and DNA damage response, and involved in the initiation and progression of human diseases. In this review, we summarize the current knowledge of lincRNA-p21, mainly focus on the known biological functions and its underlying mechanisms. Moreover, we highlight the growing body of evidences for the importance of lincRNA-p21 in diverse human diseases, which indicate lincRNA-p21 as a potential diagnostic marker and/or a valuable therapeutic target for these diseases.

## 1. Introduction

Long non-coding RNAs (lncRNAs) are generally defined as transcribed RNA molecules that are larger than 200 nucleotides (nt) and have no significant protein-coding capacity. They are RNA polymerase II (Pol II) transcripts, and thus are capped, spliced and polyadenylated [1]. The recent explosion in knowledge has revealed the critical role of lncRNAs in the regulation of multiple biological processes impacting on development, differentiation, and metabolism, and thus brought these heretofore neglected players to the forefront [2,3,4]. Nonetheless, only a handful of functional lncRNAs are represented in the human genome as yet. A growing body of evidence suggests that *lincRNA-p21*, which locates approximately 15 kb upstream of the cell-cycle regulator gene *p21/Cdkn1a* and ~3.0 kb in length, is a novel regulator of cell proliferation, apoptosis and DNA damage response, and plays an important role in the development and progression of human diseases [5,6,7]. LincRNA-p21 contains different motifs embedded in its natural higher-ordered structure. These motifs interact with RNA-binding proteins, miRNAs and mRNA targets to orchestrate auto-regulation and the expression of its target transcripts [5,8,9]. Here, we review the molecular mechanisms of lincRNA-p21 in the regulation of target gene expression, and the potential roles of lincRNA-p21 in human diseases.

## 2. Biological Functfion of LincRNA-p21

### 2.1. LincRNA-p21 and p21

LincRNA-p21 has a key functional role in regulating p21 levels. LincRNA-p21 recruits heterogeneous nuclear ribonucleoprotein K (hnRNP-K) to the promoter of *p21*, which is pivotal for the efficient binding of p53 to the *p21* promoter to initialize the transcription of *p21*, and therefore lincRNA-p21 can activate the expression of *p21* [10]*.* Dimitrova *et al*. [10] generated a conditional knockout mouse model to investigate the effects of *lincRNA-p21* deficiency and demonstrated that *lincRNA-p21* deletion could diminish *p21* expression and promoted mouse embryonic fibroblasts (MEFs) proliferation. However, Huarte *et al*. [5] and Bao *et al.* [11] didn’t detect any reduction in the expression of *p21* upon *lincRNA-p21* knockdown in MEFs. A potential explanation to the discrepancy is that complete suppression of *lincRNA-p21* is required to deregulate *p21* [11]. Additional experimentation need to clarify the issue.

### 2.2. LincRNA-p21 and Other Targets

**Figure 1:**
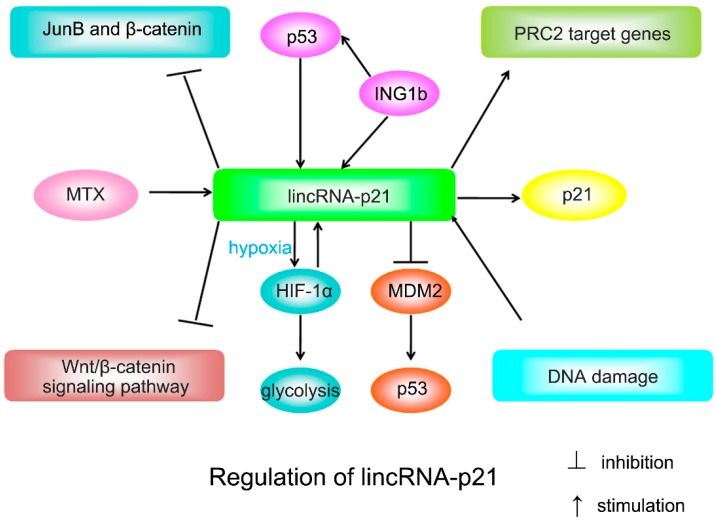
Overview of the known regulatory mechanisms for lincRNA-p21.

Mouse double minute 2 (MDM2), an E3 ubiquitin-protein ligase, can degrade p53 and block the interaction of p300 with p53, thereby inhibiting p53 acetylation and its activity [12,13,14]. p300 is an acetyltransferase that acetylates p53 to enhance its activity [15,16,17]. LincRNA-p21 can interact with several important factors including MDM2, Rck and hnRNP-K [5,6,18]. For instance, lincRNA-p21 can bind to MDM2, thus reducing the MDM2-p53 interaction and increasing p300-p53 interaction [18]. Overall, lincRNA-p21 can provide feedback to increase the transcriptional activity of p53 via binding to MDM2 and reducing MDM2-mediated inhibition of p53 (Figure 1).

**Figure 2:**
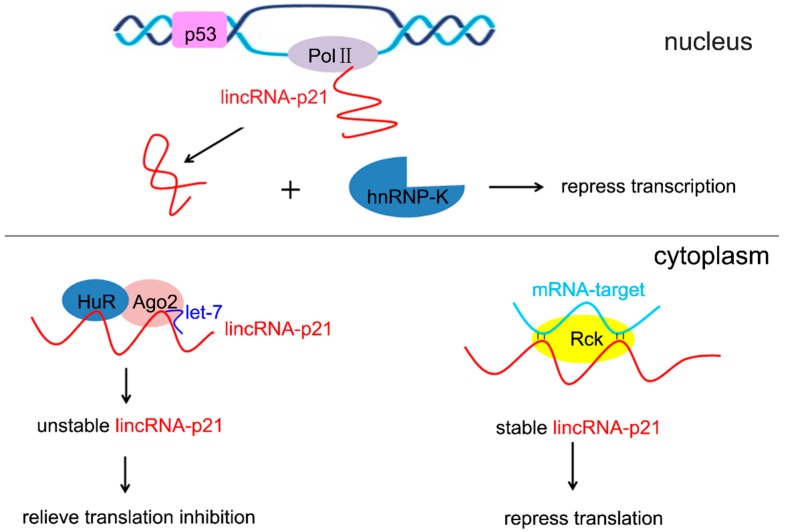
The roles of lincRNA-p21 in gene regulation. In the nucleus, lincRNA-p21 is a RNA Pol II transcript and is capped, spliced and polyadenylated. LincRNA-p21 interacts with hnRNP-K protein through a relatively conserved 5ʹ terminus and represses the transcription of target genes as part of the canonical p53 transcriptional response. In the cytoplasm, HuR is shown to interact with lincRNA-p21 and destabilize lincRNA-p21 by recruiting a let-7/Ago2 complex. LincRNA-p21 recognizes mRNA targets by basepairing and represses their translation in coordination with the Rck RNA helicase.

LincRNA-p21 possesses a post-transcriptional function as a modulator of translation. It can recognize mRNA targets by basepairing and repressing translation in coordination with the Rck RNA helicase in the cytoplasm (Figure 2). For example, lincRNA-p21 can negatively regulate the translation of CTNNB1 (β-catenin) and JUNB by the facilitation of Rck [6]. The interaction of lincRNA-p21 with CTNNB1 and JUNB mRNAs was quantified by affinity pull-down of lincRNA-p21 using biotin-labeled antisense lincRNA-p21 oligo [6]. Since lincRNA-p21 is a p53-induced lncRNA, the reductive translation of JUNB and β-catenin via lincRNA-p21 is consistent to the tumor suppressive role of p53. Hu antigen R (HuR) is a ubiquitous RNA binding protein that influences cell survival, proliferation, carcinogenesis, and immune responses. HuR can cooperate with the Ago2 protein and let-7 miRNA to destabilize lincRNA-p21, dramatically reduces its levels and relieves the translation inhibition of lincRNA-p21 target mRNAs (Figure 2) [6]. Meanwhile, heat shock factor 1-regulated HuR and let-7/miR-320 could contribute to the translation of β-catenin through down-regulation of lincRNA-p21 expression [19].

MicroRNAs have emerged as an important class of small regulatory RNAs in eliciting post-transcriptional control. The protein MS2 tagged RNA affinity purification can recognize miRNAs associated with a target transcript. Yoon *et al*. [8] found that MS2-mediated pulldown of lincRNA-p21 could identify interacting target miRNAs with functional impact on the expression of lincRNA-p21, and at least four miRNAs (miR-130, miR-221, let-7b, let-7c) were enriched in the lincRNA-p21-MS2 pulldown. It contributes to deepen our understanding of lincRNA-p21 in post-transcriptional gene regulatory schemes by MS2 tagged RNA affinity purification. LincRNA-p21 and miRNAs may form specific structures, which are involved in various regulating pathways. The relationship between lincRNA-p21/miRNAs and regulating pathways still require further study.

### 2.3. LincRNA-p21 on Somatic Cell Reprogramming

Induced pluripotent stem cells (iPSCs) have major implications for toxicology screening, regenerative medicine and disease modeling *in* *vitro* [20]. However, in order to fulfill these expectations, it is necessary to clarify how the reprogramming machinery erases and rewrites the somatic epigenetic code. LincRNA-p21 may block somatic cell reprogramming by CpG methylation at the promoters of a subset of genes (e.g., *Nanog*) and H3K9me3 maintenance at the promoters of other set of pluripotency genes (e.g., *Lin28a*), which both are mediated by hnRNP-K [11]. LincRNA-p21 contains a rather highly conserved 780 nt length 5′ region that binds with hnRNP-K [5]. The down-regulation of hnRNP-K leads to decrease lincRNA-p21 levels, suggesting that hnRNP-K is necessary for the induction and stability of lincRNA-p21 [10]. LincRNA-p21 represses genes which are downregulated as part of the p53 transcriptional response and functions at least in part by interaction with hnRNP-K (Figure 2) [5]. Some pluripotency regulators such as *Lin28a*, *Esrrb*, *Lefty2*, *Nanog* and *Sall4* are significantly upregulated by *hnRNP-K* or *lincRNA-p21* knockdown [11]. Therefore, lincRNA-p21 exerts its role in reprogramming mainly through hnRNP-K, which may constitute the essential functional unit in somatic cell reprogramming regulatory networks.

### 2.4. LincRNA-p21 on DNA Damage Response and Apoptosis

LincRNA-p21 plays a crucial role on DNA damage response. A recent study has reported that lincRNA-p21 is highly enriched in exosomes which can best reflect the increase in its expression upon DNA damage [21]. Additionally, being exposed to furan induces bidirectional transcription between *p21* and *lincRNA-p21* with dose-dependent effect [22]. Moreover, lincRNA-p21 also can regulate apoptosis under stress conditions. *LincRNA-p21* is directly induced by p53 and leads to transcriptional activation in response to DNA damage [23]. In addition, lincRNA-p21 physically interacts with hnRNP-K, confirmed by RNA-pull down, mass-spectrometry and RNA immunoprecipitation (RIP), thus regulating p53 downstream transcriptional repression and apoptosis [5]. Yang *et* *al*. [24] found that mouse lincRNA-p21 could regulate doxorubicin-induced apoptosis in MEFs. However, unlike the mouse counterpart, the human lincRNA-p21 appears not to participate in the regulation of doxorubicin-induced apoptosis [24]. Therefore, human lincRNA-p21 and its mouse counterpart are not functionally equivalent, which may be caused by the low sequence conservation of *lincRNA-p21* across humans and mice. ING1b is a tumor suppressor which affects apoptosis and cell cycle control. LincRNA-p21 is upregulated by ING1b and this effect is additive to p53 (Figure 1) [25]. In addition, lincRNA-p21 enhances the ability of ING1b to induce apoptosis [25]. Thus, lincRNA-p21 is a mediator of ING1b-induced apoptosis. Nonetheless, the precise mechanism by which lincRNA-p21 leads to apoptosis remains to be defined.

## 3. LincRNA-p21 in Diseases

### 3.1. LincRNA-p21 in Cancers

LincRNA-p21 is implicated in the development and progression of human diseases, particularly in cancer. Cancer cells exhibit a particular metabolic phenotype referred to as the Warburg effect, characterized by reduced oxidative phosphorylation and enhanced glycolysis [26]. It has been shown that activation of hypoxia-inducible factor-1α (HIF-1α) contributes to the Warburg effect under hypoxic conditions [24]. LincRNA-p21 is a hypoxia-responsive lncRNA and plays a critical role in the regulation of the Warburg effect through regulation of HIF-1α pathway. HIF-1α-induced lincRNA-p21 can bind HIF-1α and Von Hippel-Lindau (VHL), and thereby disrupts the VHL-HIF-1α interaction [24]. Furthermore, lincRNA-p21 in turn stabilizes HIF-1α, therefore forming a positive feedback loop to make sure hypoxia-induced HIF-1α expression under hypoxic conditions (Figure 1) [24]. The HIF-1α-lincRNA-p21 axis facilitates tumorigenesis, thus lincRNA-p21 may represent a therapeutic target for cancer treatment.

In addition, Zhai *et al.* [27] found that lincRNA-p21 was aberrantly expressed in colorectal cancer (CRC), and its level was associated with CRC stage and tumor tissue invasion. Moreover, lincRNA-p21 may promote the sensitivity of radiotherapy for CRC mainly through suppression of the β-catenin signaling pathway and elevation of the expression of the pro-apoptosis gene *Noxa* [28]. The Wnt/β-catenin signaling pathway is a critical pathway in CRC initiation and progression, and the β-catenin aberrant activation is harmful to CRC treatment [29,30,31]. LincRNA-p21 may inhibit Wnt/β-catenin signaling pathway through reducing the expression of β-catenin target genes, such as c-myc and cyclin D1 [28]. Therefore, lincRNA-p21 not only deepens our understanding of the mechanism of CRC carcinogenesis but also offers a potential target for CRC radiotherapy.

LincRNA-p21 also has been reported to be associated with skin tumors, prostate cancer and chronic lymphocytic leukemia (CLL) [32,33,34,35]. A recent study has revealed that down-regulated lincRNA-p21 may favor the hyper-proliferation of keratinocytes and then promote skin tumor formation in VDR null mice [32]. LincRNA-p21 promotes UVB-induced apoptosis in human and mouse keratinocytes [33]. A mutation in a single p53 allele inhibits the effect on the UVB-induced expression of lincRNA-p21 and then evades the UVB-induced apoptosis, eventually offering a pro-oncogenic function early in the development of skin cancer [33]. Therefore, lincRNA-p21 may act as a tumor suppressor in UVB-induced skin cancer. LncRNAs often display tissue- and disease-specific expression which can provide important potential biomarkers specific to the particular disease type. Isin *et al.* [35] found that the exosomal lincRNA-p21 may act as a stability and non-invasive diagnosis biomarker for the detection of prostate cancer. In addition, lincRNA-p21 is significantly down-regulated in CLL patients, tested by the real-time PCR assay (forward primer sequence: 5′-GGGTGGCTCACTCTTCTGGC-3′ and reverse primer sequence: 5′-TGGCCTTGCCCGGGCTTGTC-3′ [5]), and the decreased plasma lincRNA-p21 level correlates with the CLL stage, which showed the clinical significance of lincRNA-p21 in CLL as a potential biomaker [34].

### 3.2. LincRNA-p21 in Other Diseases

LincRNA-p21 also participates in the pathogenesis of other diseases. For instance, lincRNA-p21 is an important regulator of cell proliferation and apoptosis during atherosclerosis [18]. The expression of lincRNA-p21 is dramatically downregulated in coronary artery disease patients and atherosclerotic plaques of *ApoE*^−/−^ mice, which showed that lincRNA-p21 may act as a therapeutic target to treat cardiovascular disorders and atherosclerosis [18]. LincRNA-p21 also plays an important role in the development and progression of rheumatoid arthritis (RA). Patients with RA exhibited decreased basal levels of lincRNA-p21 and increased the levels of RelA, a marker of NF-κB activation, and then led to an increase in NF-κB activity [36]. Methotrexate (MTX) can increase the expression of lincRNA-p21 and diminish the basal levels of NF-κB in RA chemotherapy [36].

## 4. Conclusions

LincRNA-p21 is involved in diverse cellular processes via regulation of multiple target gene expression, and has been proven to be a crucial regulator in physiological processes and diseases. However, the biological function of lincRNA-p21 is still largely unknown. Further studies are required to unveil other effects of lincRNA-p21 through forming lncRNA-p21-protein/mRNA complexes and its role in the regulation of gene expression. In addition, dysregulation of lincRNA-p21 is involved in the development and progression of diverse human diseases. Therefore, whether lincRNA-p21 can be utilized as a diagnosis biomarker and/or drug target in these human diseases needs to be further investigated. It will be an exciting journey to form a better understanding of the precise lincRNA-p21 molecular mechanisms, and further explore a convenient diagnosis biomarker and/or therapeutic target for diverse human diseases. In conclusion, the potential roles of lincRNA-p21 in biomedical science could be tremendous, and will require a long time of intensive research before it can be fully deciphered and applied.
